# Stochastic simulation modeling of the economics of providing additional living space for housed dairy cows

**DOI:** 10.3389/fvets.2024.1473696

**Published:** 2024-12-05

**Authors:** Jake S. Thompson, Chris D. Hudson, Jon N. Huxley, Jasmeet Kaler, Martin J. Green

**Affiliations:** ^1^School of Veterinary Medicine and Science, University of Nottingham, Sutton Bonington, Leicestershire, United Kingdom; ^2^School of Veterinary Science, Massey University, Palmerston North, New Zealand

**Keywords:** dairy cow housing, economics, living space, simulation model, welfare

## Abstract

The housed environment for dairy cattle is of critical importance to their health, wellbeing, and productivity. Lack of space is an important factor for housing quality assessment due to links with increased likelihood of disease. A recently published randomized controlled trial identified that greater living space provision increased lying time, milk volume production, and also increased time to conception. However, despite probable improvements in cow welfare, the question remains as to whether offering increased living space is a cost-effective option for farmers. The costs associated with financing new housing facilities are escalating, and the industry urgently requires an evidence base for ensuring these investments are financially sustainable. This research used stochastic simulation modeling to explore theoretical net returns on infrastructure investment differences between two living space scenarios (3 m^2^ vs. 6.5 m^2^). A cow entered a simulation at the point of first calving, and milk production, reproductive performance, and points of exit were stochastically determined over the cow’s lifetime simultaneously based on living space scenario. This allowed for direct financial comparison over specified sets of parameter inputs. Where cows exited the herd within their second to fourth lactation, the median difference in financial return was observed to be +£87.61 per cow per year (mean + £86.74). The estimated return on investment to provide extra living space access varied dependent on provision method, interest rates, and loan repayment duration. Under the circumstances and contexts investigated, the results suggest that building for increased living space would be cost-effective. When building a new shed with a high living space versus control at a 4.00% interest rate, a median net return on infrastructure investment of +£23.00 per cow per year was identified (range –£25.91 to +£64.16 for 10th to 90th percentile). Since decreased living space is likely to lead to poorer welfare, it can be considered a negative production externality associated with current production systems, the cost of which should also be accounted for when analyzing the economics of housing. Further research is essential to gain a complete understanding of the cost-effectiveness of providing increased living space per cow under different management scenarios.

## 1 Introduction

Housing of dairy cattle is a common husbandry method used worldwide, with a trend toward year-round housed systems (1). With an increasing amount of time spent indoors, the housed environment is of critical importance to the health and wellbeing of dairy herds. Indeed, it has been shown that housing conditions impact the health and wellbeing of several species, including humans. For example, a study assessing the impact of housing in humans reported that lack of access to outdoor space and higher living densities impaired the mental wellbeing of the participants (2). Similarly, lack of space has been noted as the most important factor when housing quality is assessed due to its links with increased likelihood of disease (3, 4). Although similar research in farmed animals is limited, it has recently been shown in a randomized controlled trial involving housed dairy cows that a simple increase in living space results in increased milk production, increased lying times, and decreased idling in passageways but a poorer reproductive performance (5). For dairy cows, however, despite probable improvements in cow welfare, the question remains as to whether offering increased living space is a cost-effective option for the farmer.

In terms of farm economics, increased milk volume production efficiency is generally associated with improved profitability (6, 7), whereas poorer fertility (via increased time to conception) is associated with reduced profitability (8). Increased time to conception has been associated with negative impacts on financial returns due to cows being delayed in return to peak lactation (43). The results from the only study on living space in dairy cows (5) revealed that additional space led to increased milk yield but poorer fertility, but the financial impact of these differing effects was not evaluated. The purpose of this study was to explore and understand the complexity of this relationship to determine whether provision of additional space for dairy cows is likely to be cost beneficial.

This question is particularly pertinent since herd sizes are generally increasing especially with regards to farms with higher yielding cows which usually require indoor housed environments (1). This has led to a need to invest in new housing infrastructure, commonly in the form of freestall accommodation, and the industry requires an evidence base to ensure that a large capital investment results in a sustainable and financially secure farm business. An investment in farm infrastructure should aim to create an optimal environment for dairy cow welfare as well as a financial return for farmers.

Simulation modeling methods are commonly adopted when many potential outcomes or scenarios require investigation (9–11), meaning experimental methods are unfeasible. Therefore, simulation models are useful for first line investigations to establish broad patterns and, through stochasticity of input parameters, establish key components that drive a model. Such models have been commonly used in the dairy industry, for example, to assess the potential financial implications of herd management practices to herd reproductive performance (12, 13) and optimize cow flow design for robotic milking sheds (14, 15).

The aim of this research was to construct a stochastic simulation model to explore the possible financial impacts (including return on investment) of providing additional living space for dairy cows. Based on previous research (5), we considered two specific living space scenarios in which the cows were provided with either 3 m^2^ or 6.5 m^2^ per cow of living space (equivalent to a total space allowance of 9 m^2^ or 14 m^2^ per cow, respectively).

## 2 Materials and methods

Model building was undertaken in R statistical software, version 3.6.1. R packages tidyverse (16), lme4 (17), and minpack.lm (18) were used. Ethical permission for this research was granted by the UK Home Office and the University of Nottingham Ethical Review Committee (license number MG_P07992717).

### 2.1 Purpose and outline structure of the model

The stochastic simulation model was structured to allow a financial comparison to be made between two living space scenarios (living space: 3 m^2^ vs. 6.5 m^2^ per cow, with total space: 9 m^2^ vs. 14 m^2^ per cow), based on differences in cow performance previously reported for Holstein dairy cows in year round housed and milked via an automated milking system (5). The purpose was to identify the variation in net return on investment dependent on the infrastructure required to provide increased living space. Living space has been defined as the area considered to be above a baseline minimum required for movement and feeding in dairy cow accommodation, excluding lying space areas (19). Total space has been defined as all floor and bedded areas that are accessible to dairy cows, including stalls, all passageways, and loafing spaces (19).

A cow entered the simulation at the point of first calving, and milk production, reproductive performance, and herd exit events were simulated over the cow’s potential lifetime. The trajectory of each cow was simulated simultaneously through both living space scenarios, which allowed for a direct financial comparison to be made for specified sets of parameter inputs. Based on previously published research, differences between the two simulated living space scenarios were assumed to arise from (i) differences in the level of milk production per cow and (ii) differences in the time to conception per cow (5).

The length of a cow’s life was defined by a time to exit from the herd based on parity of exit and the length of the final lactation which was given stochastic variation (*n* = 5). For each simulated pair of cows, the parity of exit was pre-determined to occur for every parity between 1 and 10. Therefore, the effect of parity at exit was explored equally across all 10 parities, with the addition of five final lactation lengths (*n* = 50 simulations per matched pair).

By incorporating stochastic parameters for milk yield, reproductive performance, and milk price, expected financial returns from each cow’s lifetime were estimated. Variable costs (such as mean associated cost of purchased feed and cost risk associated with transition period) between the two scenarios were subtracted from the total expected revenue to provide a financial return difference per cow lifetime between matched living space scenarios. The two living space scenarios were compared as a financial difference per cow place in the herd and as a financial return per cow per year. A large number of simulations (*n* = 200,000 per parity of exit; *n* = 2,000,000 total) over realistic input domains were conducted for randomly generated cows with stochastic variability in the main model parameters. Having completed the stochastic simulation, a regression model was constructed to assess the impact of each model parameter on the expected net return on infrastructure investment, dependent on living space provision.

**Figure 1:**
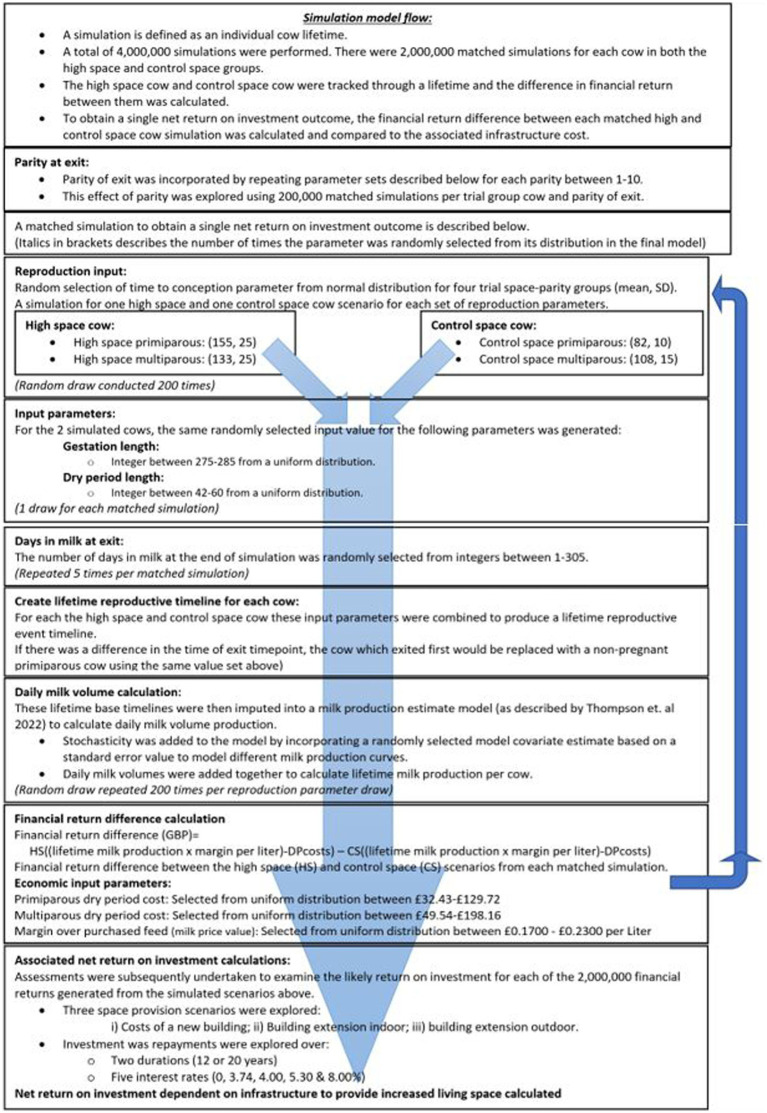
Flow diagram to illustrate the simulation model for calculating the predicted net return on investment dependent on living space provision. A simulation runs through a single cow lifetime from point of first calving. Stochastic input variables and number of sampling events are detailed where imputed and include time to first conception, milk yield modeling, parity at herd exit, gestation length, dry period length, and duration of final parity. These inputs then create a scenario timeline for a high space and control space cow, respectively.

The structure of the simulation model is illustrated in Figure 1, and further details of individual model components are described below.

### 2.2 Model setup, input variables, and build

The stochastic model incorporated probability distributions that described the time to conception analysis and milk production curves, as defined in Thompson et al. (5). In summary, this previous research comprised a randomized, controlled [1:1], long-term (1 year), longitudinal, parallel-group (pairs of adult dairy cows matched by parity and days in milk), cross-over (group location within facility) study to evaluate superiority/inferiority of a space allowance intervention for housed dairy cattle (5). The distributional assumptions used for milk production and time to conception are described below.

#### 2.2.1 Reproductive parameters

Reproductive performance was represented by time to conception, which was drawn for each cow from normal distributions based on the findings of Thompson et al. (5). A time to conception in days for each cow parity was drawn from one of the following four normal distributions (mean, standard deviation): control space primiparous (82, 10), high space primiparous (155, 25), control space multiparous (108, 15), and high space multiparous (133, 25). The multiparous parameter remained the same for each parity greater than two in that simulation.

Further stochastic input parameters were used as follows:

- Gestation period duration: This was selected from a uniform distribution between 275 and 285 days (20).
- Dry period duration: This was selected from a uniform distribution between 42 and 60 days [based on typical on-farm management (21)].

#### 2.2.2 Milk yield parameters

Milk yield was estimated daily for each cow throughout its lifetime. Daily yield was predicted from the mixed effects model described by Thompson et al. (5), which incorporated living space scenario, days in milk, parity category (primiparous or multiparous cows), and reproductive status (non-pregnant or five gestation categories) to predict lactation milk curves. Stochasticity was incorporated by drawing from the parameter distributions of the model covariates to simulate different milk production curves for each cow lifetime. An example of the resulting variability in milk yield curves is provided in Supplementary Figure S1.

#### 2.2.3 Determination of a cow lifetime: exit of cow from the herd

A cow lifetime was defined by specifying the number of calving prior to exit and the number of days in milk at exit in the final parity. Therefore, once milk yield and reproductive parameters were allocated for a simulated cow (repeated 40,000 times per trial group), that cow was used in 50 separate simulations (a simulation for an exit in each parity between 1 and 10 with each parity having 5 possible exit times) for each living space group. A total revenue was calculated for each cow lifetime. When there was a time difference between living space groups in a cow being culled (i.e., the end of the final lactation did not coincide temporally), the cow culled earliest was immediately replaced by a first primiparous cow with the same milk yield and reproductive parameters, until the culling timepoint was reached for that scenario.

Therefore, parity of exit was explored equally for all input parameter sets for each parity of exit between 1 and 10. For each parity of exit, a simulation was conducted such that a cow could exit at one of five times between 1 and 305 days in milk; these five exit times were drawn from a uniform distribution (1, 305). A total of 200,000 simulations were conducted per parity of exit per trial group resulting in 2,000,000 simulated comparisons being made between trial group. The visual representation of the simulation model is provided in Figure 1.

### 2.3 Partial budget for living space scenario comparisons

For the purposes of this research, the difference in “financial return” between the two living space scenarios was defined as the simulated difference of income per cow per year over purchased feed between a cow provided a high versus a control living space environment (Equation 1). This was defined as the cost of milk minus the mean associated cost of purchased feed required for the level of production. Only economic values that were necessary to calculate the difference in financial return between the two living space scenarios were used in the model; all other costings (inputs and outputs) assumed to be the same for both groups. Values that were not deemed to be directly dependent on living space provision (e.g., income from a cull cow sale) were assumed to be identical between scenarios and thus not included in the simulation.


(1) $$\begin{array}{l} \mathrm{Financial}\;\mathrm{return}\;\mathrm{difference}\;\left(GBP\right)\\ =HS\left(\left(\mathrm{lifetime}\;\mathrm{milk}\;\mathrm{production}\;x\;\mathrm{margin}\;\mathrm{per}\;\mathrm{liter}\right)-\mathrm{DPcosts}\right)\\ -CS\left(\left(\mathrm{lifetime}\;\mathrm{milk}\;\mathrm{production}\;x\;\mathrm{margin}\;\mathrm{per}\;\mathrm{liter}\right)-\mathrm{DPcosts}\right) \end{array}$$

The calculation used to determine financial return difference between the high space (HS) and control space (CS) scenarios from a single simulation. Dry period (DPcosts) is the economic cost associated with disease risk following a dry period. GBP means Great British Pounds.

The predicted daily milk yields were cumulatively added to produce an estimate of the lifetime production of a simulated cow lifetime. The margin per liter of milk over purchased feed was calculated using the published mean in the UK, which was identified as £0.2049 per liter (22). Stochasticity to milk margin was included by allowing the simulation model to draw from a uniform distribution between £0.1700 per liter and £0.2300 per liter. The margin per liter of milk was set to be the same for both trial groups for each simulation.

#### 2.3.1 Transition health events

Since a key difference between the living space scenarios being considered was duration of productive life, the number of calving events in a lifetime may be different between the two scenarios. Thus, the costs associated with a dry and transition period were incorporated in the models. The cost of a transition period was calculated using data from Liang et al. (23) for common transition diseases (metritis, mastitis, ketosis, lameness, and dystocia) based on stochastic modeling. These costs per disease incident originating from the transition period were averaged to a risk per cow basis using mean disease incidences for UK dairy farms (24). To add stochasticity to this variable, the transition cost was drawn from a uniform distribution *U*(32.43–129.72) for primiparous cows and *U*(49.54–198.16) for multiparous cows, the possible range being based on halving or doubling the estimated costs of £64.86 per primiparous cow per transition and £99.08 per multiparous cow per transition. These figures were inclusive of the death risk associated with transition cow disease and thus incorporated the financial aspect of cows being at higher risk of death at this time.

### 2.4 Regression model to assess impact of model input parameters on financial return

To evaluate the impact of each model input variable on the financial return per cow per year, a linear regression model was built, Equation 2. All variables used in the model were centered and standardized (subtracted from the mean and divided by the standard deviation). One variable was omitted, “lifetime days,” due to its close correlation with another variable, “number of lactations at exit.”

A forest plot was utilized to illustrate the influence that each input variable had on the financial return difference per cow per year. The regression model took the form:


(2) Yi=β0+βiXi+εi,

Where subscript *i* denoted the *i*^th^ simulated comparison between group, *Yi* the financial return difference per cow per year for simulation *i*, and *β0* the intercept, *Xi* signified a matrix of input variables included in the final model, *β* signified the coefficient related to each input variable, and *εi* was the residual error, assumed to be normally distributed with mean = 0 and variance σ2.

### 2.5 Net return on investment dependent on infrastructure used to provide increased living space

A calculation was made for the building costs associated with providing extra living space for lactating dairy cows (i.e., for the difference in space provision between the high and control space scenarios), termed net return on investment herein. Assessments were made for additional space provided as either a new building or through extension of an existing building. The associated costs displayed in this section have been adjusted based on inflation rates published by the UK Office of National Statistics Construction output price indices (25).

#### 2.5.1 Costs of a new building

To estimate the cost of building a new housing facility, with varying space provision, the AHDB housing wizard calculator was used (44). Total floor space allowances of 9 and 14 m^2^ per cow were used to estimate costs. A scenario was set using a 100-cow herd housed for 365 days per year bedded on sand cubicles; this estimate was for a ‘2-cubicle row with feed-face’ system. The cost of systems based on 2 rows or 3 rows of cubicles per row of feed face appeared similar between estimates; therefore, the costing of the 2-row system was used as a comparator. The inputs used were based on costings for the building shell and flooring; this was set at £182.00 per m^2^. This estimate does not include cubicle or other internal infrastructure costs, but these were assumed to be the same between trial scenarios, as the number of animals remained the same and only total floor area varied.

Capital investments are often repaid over prolonged time periods, and the cost of these investments was converted to an expected annual repayment with a fixed loan interest rate set at 4.00%, based on the median UK rate of the last 25 years. These repayment costs were compared to the valuations from the final financial returns model to estimate an approximate net return on infrastructure investment. For comparison, fixed loan interest rates of 0.00, 3.74, 5.30, and 8.00% are shown in Table 1.

**Table 1:** Results of a regression model assessing the stochastic parameter inputs to estimate the difference in financial return between cow lifetimes simulated in control living space (3 m^2^) versus high living space (6.5 m^2^) housing.

Predictors	Estimates	95% Confidence Interval	*p*-value
(Intercept)	0.083	0.080–0.087	
Parity exit number (reference = 3)			
One	1.18	1.17–1.18	**<0.001**
Two	0.52	0.51–0.52	**<0.001**
Four	−0.20	−0.20 – −0.19	**<0.001**
Five	−0.30	−0.30 – −0.29	**<0.001**
Six	−0.35	−0.36 – −0.35	**<0.001**
Seven	−0.39	−0.39 – −0.38	**<0.001**
Eight	−0.41	−0.42 – −0.41	**<0.001**
Nine	−0.43	−0.44 – −0.43	**<0.001**
Ten	−0.44	−0.45 – −0.44	**<0.001**
Milk production model standard error deviation of estimate parameters	−0.040	−0.041 – −0.039	**<0.001**
Milk price (GBP)	0.12	0.12–0.12	**<0.001**
Cost primiparous dry period (GBP)	0.0002	−0.0009 – 0.0014	0.73
Cost multiparous dry period (GBP)	−0.0009	−0.0021 – 0.0002	0.11
Primiparous high space days non-pregnant	0.022	0.021–0.024	**<0.001**
Primiparous control space days non-pregnant	−0.017	−0.019 – −0.016	**<0.001**
Multiparous high space days non-pregnant	0.17	0.17–0.17	**<0.001**
Multiparous control space days non-pregnant	−0.054	−0.055 – −0.053	**<0.001**
Dry period duration	−0.054	−0.055 – −0.053	**<0.001**
Gestation duration	0.023	0.022–0.024	**<0.001**
Final lactation duration	−0.023	−0.024 – −0.022	**<0.001**
Observations	2,000,000		
*R*^2^/*R*^2^ adjusted	0.306/0.306		

GBP is the abbreviation for unit of currency in Great British Pounds. Bold values indicate a significant *p*-value <0.05.

#### 2.5.2 Building extension modification

Two approaches were considered to modify current housed infrastructure by providing additional living space from a 3 m^2^ per cow living space allowance to a 6.5 m^2^. The cost for increasing the area for both modifications was calculated for a 100-cow herd. The capital investment required was converted to an expected annual repayment, as described for construction of a new building above.

The first modification was a building extension to provide an indoor “loafing area” (an open area of concrete) to increase living space allowance. An AHDB housing report (44) stated that a general-purpose steel frame, roofed building with a concrete floor would cost £248 per m^2^. This cost appeared to be greater on a square meter basis to a new build due to the complexity involved with shed modification in terms of removal of part of existing infrastructure to replace with new materials to allow for joining of the existing building with new extension.

An alternative method of increasing living space from 3 to 6.5 m^2^ was to add 3.5 m^2^ of additional outdoor “loafing area” (an open area of concrete). The Agricultural Budgeting and Costing book for UK agricultural buildings (42) provides an indicative cost of a fenced, concrete-based, outdoor area, to be in the region of £100.75 per m^2^.

#### 2.5.3 Sensitivity analysis: interest rates and loan repayment period

For each building scenario investigated, the investment required was tested through sensitivity analysis. Each scenario was tested using interest rates of 0, 3.74 (quartile 1), 4.00 (median), 5.30 (mean), and 8.00 (quartile 3) percent APR over a loan repairment period of 12 or 20 years. Interest rates tested were determined by calculating the interquartile range, mean, and median interest rates of the Bank of England Official Monthly average UK interest rate of loans, on a fixed rate to small- and medium-sized enterprises (in percent) not seasonally adjusted, between January 1998 to December 2022 (26, 27). Each of these costs was compared to the average financial return obtained from the simulation model for 2–4 lactations (28, 29) and the UK average cow lactation of 3.7 (30), to estimate the likely return on investment due to increased milk volume production and reduced time to conception for cows inhabiting the higher living space scenario, dependent on the cost difference to provide the extra space.

## 3 Results

### 3.1 Simulation model of financial return associated with living space

**Figure 2:**
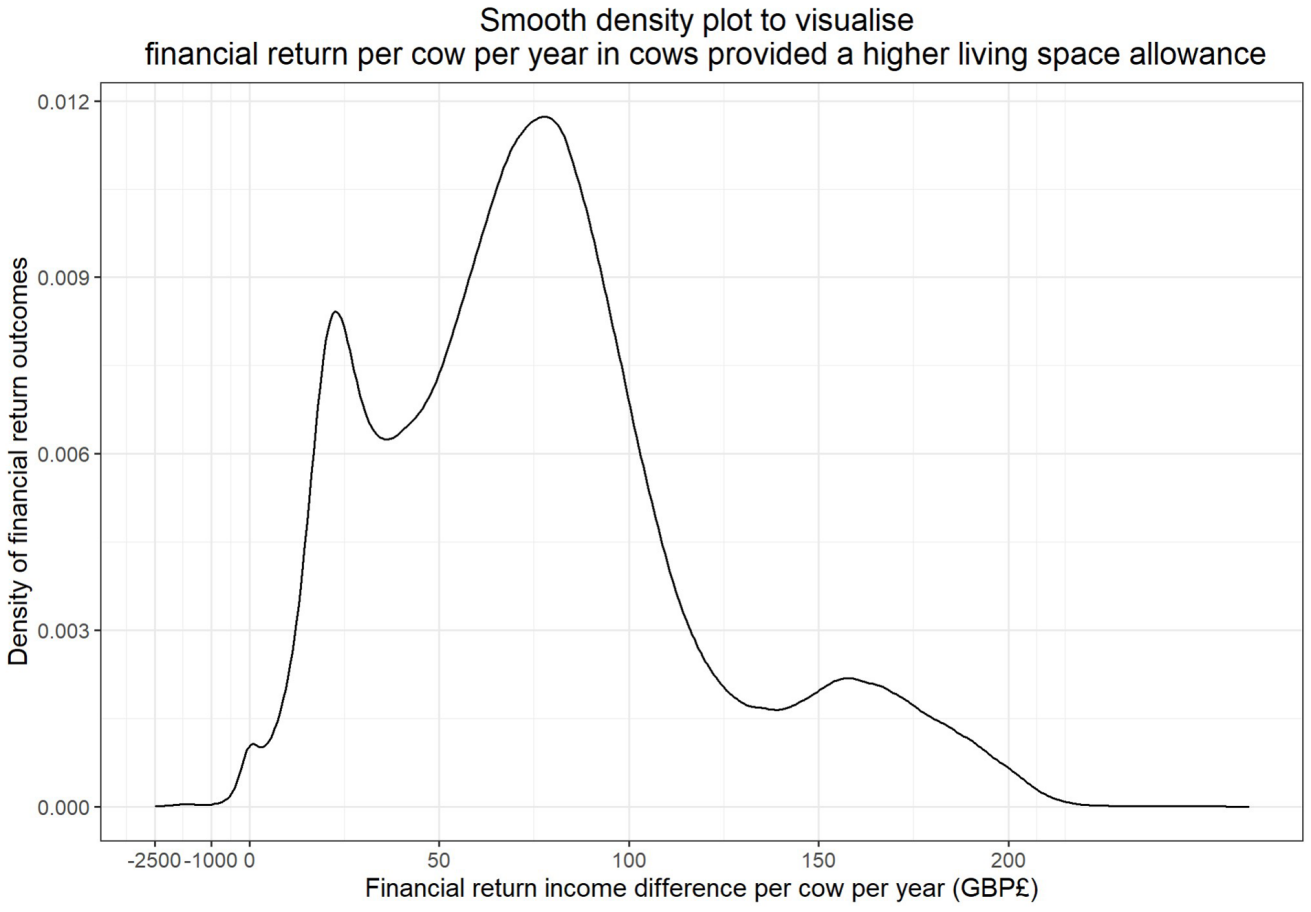
Smoothed density plot to illustrate the distribution of financial return difference per cow per year (£) for the 2,000,000 scenarios simulated to compare the economic impact of providing a high space (14 m^2^) housed environment versus control (9 m^2^). NB. The *x*-axis has been scaled differently at <£0 to represent the tail of the distribution.

The results of the simulations to explore the difference in financial return between living space scenarios dependent on different stochastic parameters are shown in Figure 2. Across 99.58% scenarios tested through the simulation model (1,991,551/2,000,000), herds with increased living space had superior financial returns.

**Figure 3:**
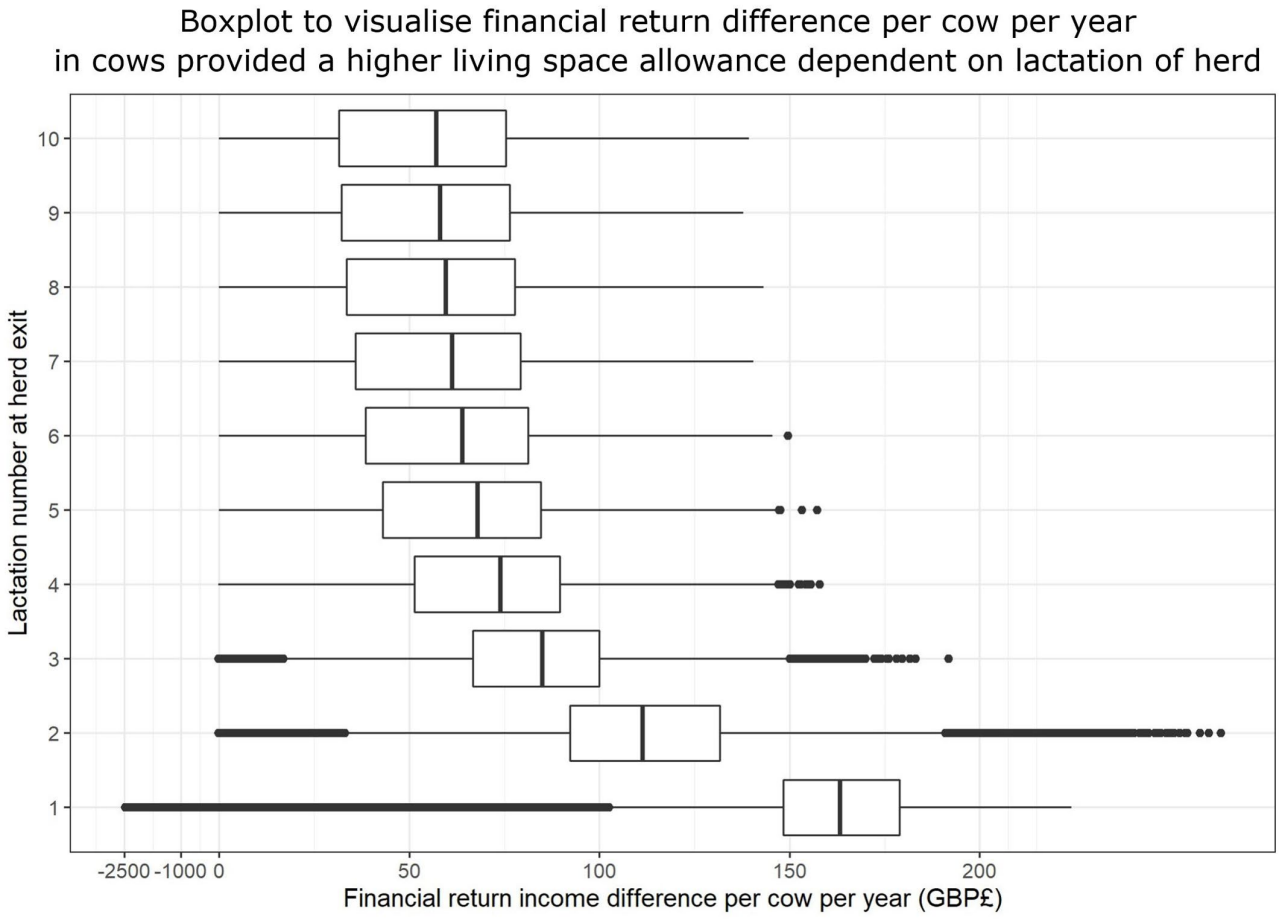
Boxplots to illustrate the distribution of financial return difference per cow per year (£) for the 2,000,000 scenarios simulated to compare the economic impact of providing a high space (14 m^2^) housed environment versus control (9 m^2^) dependent on the number of lactations at herd exit. NB. The data before £0.00 have been scaled due to tail.

In high yielding dairy herds mainly using Holstein genetics (31), an average cow will exit the herd after 2.5–4 lactations (28, 29). The results from this model suggested that cows in the high space group would rarely be associated with an inferior financial return compared to the control space (2,056/600,000 simulations or 0.34%), when exiting the herd between their second and fourth lactation, as shown in Figure 3. The summary statistics for this population indicate that the high space group have a superior median financial return of £87.61 per cow per year (interquartile range £65.59–£107.51). The median lactation number at exit for UK dairy cows is currently 3.7 (30); the model identified that for such cows, the high space group have a superior median financial return of £84.62 per cow per year (interquartile range £66.47–£99.42).

### 3.2 Regression model to assess the impact on model input parameters on the financial return per cow per year dependent on living space provision

**Figure 4:**
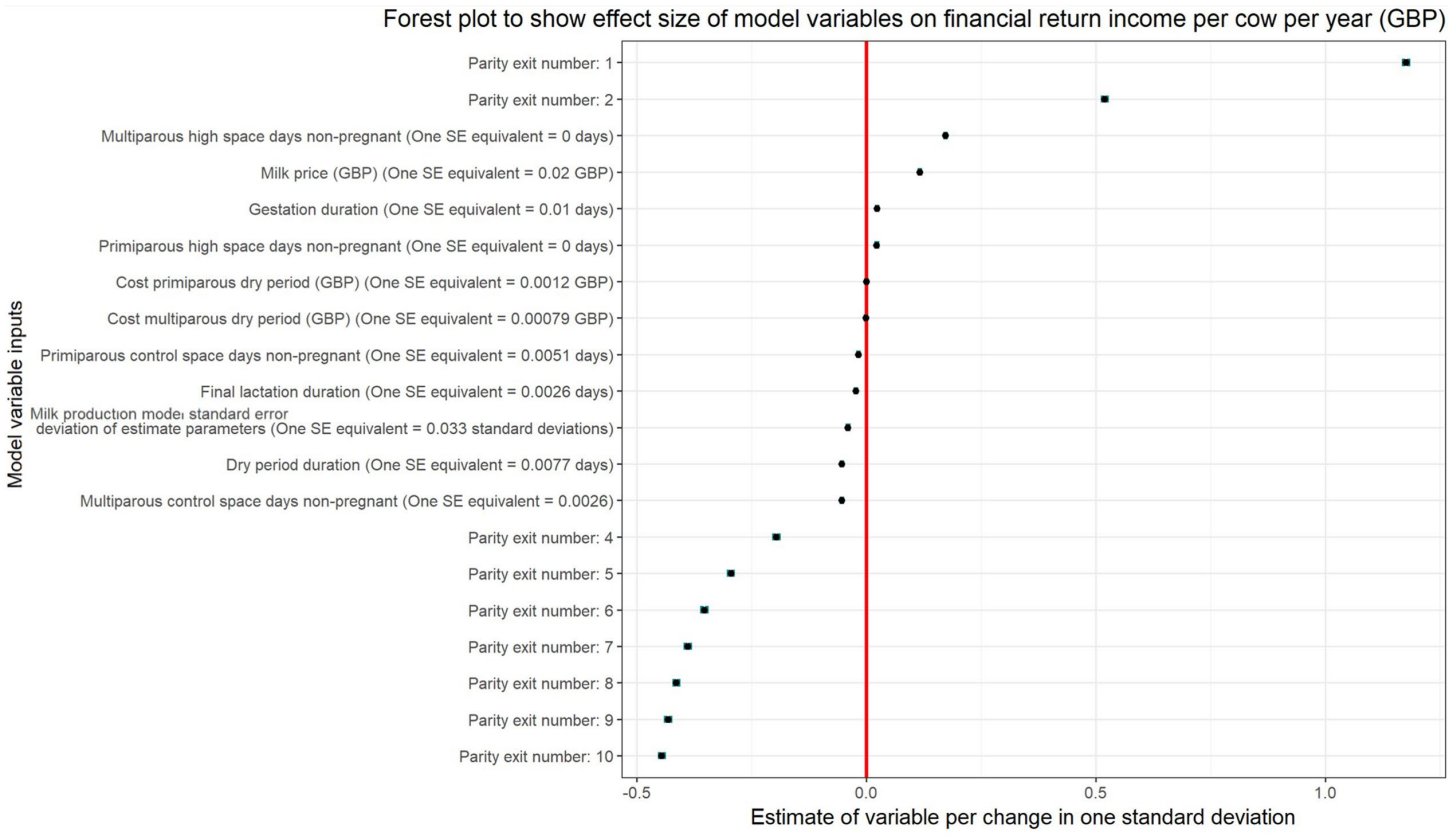
Plot shows the estimate of variable per change in one standard deviation based on the regression model to explore the effect size of model variables on financial return (income per cow per year in GBP). The greater the distance of the square dot away from the red line, the larger the influence on financial return output. Parity exit number was categorical with a reference category of 3. Brackets next to continuous variables provide detail on what each standardized estimate relates to a standard deviation of actual value.

To assess the impact of model input parameters and their variability, a linear regression model was used, and the results of this are provided in Table 1. The model and forest plot (Figure 4) showed that the key influences on financial return per cow per year were the parity of exit, milk price margin, and days to conception for multiparous cows.

### 3.3 Net return on investment

The following sections detail the headline net return on infrastructure investment figures from the economic simulation model against the cost of providing this additional space in three different scenarios (new-build, existing housing modification indoor, and existing housing modification outdoor). Further detail of the results can be seen in Table 1. A larger table is provided in the Supplementary Table S1 to present a wider selection of associated costings across these scenarios.

#### 3.3.1 New-build costs

The most expensive scenario for providing cows with extra living space would be to build a new building for the herd. Based on the AHDB wizard housing calculator tool, to build a new shed for 100 cows with a total space allowance of 14 m^2^ versus 9 m^2^ would cost £254,800 vs. £163,800, respectively. If this was paid with no interest added, the cost over a 20-year repayment period would equate to £45.50 per cow per year.

If loan repayments were necessary, at a 4.00% interest rate, it would cost a farm a total of £361,816 (14 m^2^) or £232,596 (9 m^2^), a difference of £129,220. This difference equates to £1,292.20 per cow space or £64.61 per cow per year. In terms of net return on infrastructure investment when building a new shed with a 4.00% interest rate, based on the estimates from the simulation model for a cow remaining in a herd between 2 and 4 lactations, this would equate to a median net return on infrastructure investment of +£23.00, with a range of −£25.91 and +£64.16 between the 10th and 90th percentiles, respectively, or a probability of a positive net return on infrastructure investment of 75.73% (cow exit within their third lactation equated to 10th percentile: −£27.39; median: +£20.01; 90th percentile +£48.13 per cow per year, probability of positive net return on infrastructure investment 76.53%).

#### 3.3.2 Existing housing modification comparison

For modifying existing buildings with extra indoor space, the cost was estimated to be £248 per m^2^; using a 4.00% fixed interest rate, this equates to £1,232.56 per cow in total or £61.63 per cow per year over 20-year loan repayment period. The median net return on infrastructure investment for this space based on the simulation model was +£25.98 per cow per year, with a dispersion of −£22.93 and +£67.14 (10th and 90th percentiles, respectively), and a probability of positive net return on infrastructure investment of 78.65% when cows exited the herd within their second to fourth lactation (Table 2).

**Table 2:** Sensitivity analysis of economic inputs: table of expected margins for different interest rates and loan repayment periods if bank borrowing is required to make capital investment into farm buildings to increase living space.

	Interest rate (%)	Total cost difference per cow	Cost difference per cow per year	Net return on infrastructure investment per cow per year based on exit within 2nd–4th lactation			
				10th percentile	Median	90th percentile	Probability of positive net return on infrastructure investment
New build shed build cost difference	0	£910.00	£45.50	-£6.80	£42.11	£83.27	86.88%
	3.74	£1267.36	£63.37	-£24.67	£24.24	£65.40	76.63%
	4.00	£1292.20	£64.61	-£25.91	£23.00	£64.16	75.73%
	5.30	£1416.42	£70.82	-£32.12	£16.79	£57.95	70.40%
	8.00	£1674.40	£83.72	-£45.02	£3.89	£45.05	55.27%
Indoor living space area extension	0	£868.00	£43.40	-£4.70	£44.21	£85.37	87.89%
	3.74	£1208.86	£60.44	-£21.74	£27.17	£68.33	78.63%
	4.00	£1232.56	£61.63	-£22.93	£25.98	£67.14	77.84%
	5.30	£1351.04	£67.55	-£28.85	£20.06	£61.22	73.39%
	8.00	£1597.12	£79.86	-£41.16	£7.75	£48.91	60.29%
Outdoor living space extension	0	£352.63	£17.63	£21.07	£69.98	£111.14	97.07%
	3.74	£491.10	£24.56	£14.14	£63.05	£104.21	95.08%
	4.00	£500.73	£25.04	£13.66	£62.57	£103.73	94.93%
	5.30	£548.86	£27.44	£11.26	£60.17	£101.33	94.13%
	8.00	£648.83	£32.44	£6.26	£55.17	£96.33	92.41%

This describes the net return on infrastructure investment for the three building scenarios to provide extra living space. This provides details of sensitivity analyses dependent on interest rates from 0 to 8.00% and loan repayment over a 20-year period. The results in green show scenarios where margin for investment to increase living space is positive for a farm. (nota bene). Once the loan repayment period has finished, the investment has been paid off and the estimated net return on infrastructure investment post this period would not be required to be offset against the initial investment costs thereafter.

For a scenario where provision of extra space was executed using an outdoor (uncovered) area, published figures suggest a cost of £100.75 per m^2^. To provide 3.5 m^2^ extra living space, this would cost £25.04/cow/year with interest payments at 4.00% over a 20-year loan repayment period. The median net return on infrastructure investment for this scenario was calculated to be +£59.58, with a dispersion of +£12.18 and +£87.70 (10th and 90th percentiles, respectively) when a cow exits a herd during their second to fourth lactation and a probability of positive net return on infrastructure investment 94.93% (Table 2).

Further details of net returns of infrastructure investment can be viewed in Supplementary Table S2, detailing cows exiting the herd in their third lactation and loan repayment periods.

## 4 Discussion

The aim of this study was to construct a stochastic simulation model to explore the variation in net financial return of providing additional living space for dairy cows over a 12- or 20-year time period. Based on the model specifications and assumptions used, the outputs suggested that provision of additional space would lead to an increased net return on infrastructure investment across the range of plausible input scenarios investigated. This provision of increased living space is therefore likely associated with being directly financially cost-effective.

Exploration of the net return on infrastructure investment of the two living space scenarios identified that duration of loan payments, interest rates, and method of increasing space were crucial in determining the expected return/loss. Providing the additional living space using an uncovered outdoor area is most likely to support a greater return on investment, compared to the cost of building a new shed with a greater space allowance or providing a covered loafing area. However, it should be noted that this type of space is different to the one explored in the original research by Thompson et al. (5), and thus, it is uncertain whether outcomes in terms of milk yield and reproductive performance would be consistent with that study. A point of interest also relates to the time duration of loan repayments as a farm business will account for investments depending on the future planning of the farm. This could affect the perceived cost benefit of the infrastructure change dependent on whether this is based on the 12- or 20-year period explored in this research.

While examining financial aspects of the provision of additional living space, it is important to consider potential for wider benefits. Although not tested in this research, it is plausible that increased living space would result in both additional cow health benefits not included in the simulation model, such as reduced incidence rates of mastitis, lameness, and infectious disease. Furthermore, increased living space has been reported to change the behavior of cows resulting in increased daily lying times of over 1 h (5), which is deemed to be beneficial (32). Other research has reported that cows will make effective use of extra space (33, 34); they are likely to become fitter (35, 36) and be in less direct competition with each other for resources (34, 37). Therefore, there may be important health and welfare benefits associated with increased living space that are as relevant to cow wellbeing and farm sustainability that are not captured in a simple net return on investment calculation.

The market for milk, such as many agricultural products, has characteristics of perfect competition; there are many sellers with negligible market share, and the product is largely undifferentiated. As a result, milk price is set by the law of supply and demand; farmers are price takers, and they have no influence over the amount they receive for their produce. In this type of market, there is a strong disincentive for individual producers to take any action which raises their cost of production, for example, by investing in more living space, as they will simply make themselves less competitive. Consequently, the poorer health and welfare outcomes associated with lower areas of living space in current milk production systems can be considered a ‘negative production externality’. A negative production externality is an undesirable and usually unintended side effect of the production of a good or service and, in this case, is the moral burden of poorer animal welfare borne by society. A detailed description of how negative production externalities can be resolved is outside the scope of this study, but it is acknowledged that in perfectly competitive markets, it requires market interventions, usually by government. Of course, the alternative is that producers or retailers (through incentivization) can try to escape the perfectly competitive marketplace by differentiating their product, for example, by selling “higher welfare” milk from cows with greater living space (38). While products such as this do exist, they have failed to gain significant traction, especially for negative production externalities such as poor welfare, which for many consumers remains out of sight and therefore out of mind. Of course, one could also conclude that many consumers are aware and simply do not care enough to pay the higher costs associated with improved welfare.

We have reviewed many of the core arguments associated with the intensification of dairy farming previously (39). Over a decade on, little has changed, and we reaffirm that these problems will only be solved if the cost of improved welfare is recognized and incorporated into the milk price received by producers, so they afford the production costs of higher welfare systems. While it is difficult to demonstrate that the cost of providing increased living space is always cost-effective based on production benefits alone, in most scenarios tested it may well become cost-effective, if the cost of poor welfare is also accounted for when analyzing the economics of housing.

A limitation of our simulation model is that conclusions are based on a variety of important assumptions and may not be generalizable to all dairy herds or systems. With any theoretical model, there are limitations to be aware of, in this case, from the inputs used and the assumptions made. Key assumptions of note were as follows: the variation around the expected margin over purchased feed per liter of milk which was found to be at £0.2049 per liter (stochastic variation set between £0.1700 and £0.2300 per liter); the transition cost estimates were realistic; there were no extra (unknown) effects of space that may have had a financial impact; and replacement of culled cows with a newly calved primiparous cow was immediate. The financial values used in the model were all based on published financial data at the time of the study and therefore directly relate to the contextual scenarios of the original space allowance trial. In addition, the value of the fixed margin over purchased feed cost was assumed to remain static over time. The value used was the mean over a long period which therefore incorporated fluctuations over a prolonged duration of time. The output of the model suggested that even when milk price is not optimal (at the lower end of the parameter stochasticity), providing cows with greater living space would lead to increased financial returns. It should be noted, however, that there have been large fluctuations in the market since this period and there will also be variations between countries. For the model outputs to be generalizable, model inputs and assumptions would need to apply to different herds with different systems and yields; whether this is realistic remains unknown. Further research would help to establish whether the model inputs were appropriate for other systems.

The lactation number at exit, milk price margin (increased financial return difference due to greater difference in income per liter of production between living space scenarios), and time to conception in multiparous cows were found to have the greatest influence on the financial return. Culling decisions on farm and reason for exit from the herd have been described as one of the most important in terms of overall farm profitability (28, 40). This is important in this context because a large proportion of dairy cows are culled prior to the end of their third lactation and lactation number when culling occurs, milk yield and days of productive life are key drivers of farm economic success (41). Interestingly, the simulation model identified that financial return per cow per year generally showed a greater positive difference for high space cows when cows were culled earlier from the herd. This suggests that if a cow were to exit in an earlier lactation number, they would be less economically costly if provided with a higher living space compared to if provided with control space conditions. To provide an example, a high space cow would return +£99.20 per year on average compared to a counterpart in the control space group if they exited the herd in their first lactation, whereas a cow exiting in their fifth lactation would have a return difference of +£8.54 in favor of the high space conditions. These results raise the possibility that if a farm was unable to provide additional space for the whole herd, it may be that the provision of additional living space solely for primiparous cows could provide a cost-effective return on investment. However, this hypothesis was not directly investigated, so caution is needed before extrapolating results to different scenarios.

In conclusion, this simulation model presents evidence that the provision of additional living space is associated with an increased net return on investment, which is dependent on type and repayment of infrastructure, average lactation number at herd exit, and milk price margin.

## Data Availability

The data analyzed in this study is subject to the following licenses/restrictions: not available due to commercial sensitivity. Requests to access these datasets should be directed to Jake S. Thompson, jake.thompson2@nottingham.ac.uk.

## Supplementary material

SUPPLEMENTARY FIGURE S1 Figure to show the stochastic variation added to the milk volume production models. As the standard of error increases from negative (dark blue) to positive (light blue) the overall production curve of the simulated cow daily milk production increases (*y*-axis) and this is also dependent on days in milk (*y*-axis). This has been split by effect to high space versus control and primiparous versus multiparous lactation curves.

SUPPLEMENTARY TABLE S1 Sensitivity analysis of economic inputs: table of profits/losses for different interest rates and loan repayment periods if bank borrowing is required to make capital investment into farm buildings to increase living space. This describes the net return on infrastructure investment for the three building scenarios to provide extra living space. This provides details of sensitivity analyses dependent on interest rates from 0 to 8.00% and loan repayment over a 12 and 20-year periods for cows exiting the herd while within their 2–4th lactation and 3rd lactation only. Results in green show scenarios where investment to increase living space is more profitable for a farm. N.B. Once the loan repayment period has finished the investment has been paid off and the estimated net return on infrastructure investment post this period would not be required to be offset against the initial investment costs thereafter.
