# Supplementary figures and images for: Stochastic simulation modeling of the economics of providing additional living space for housed dairy cows
Source: Front Vet Sci. 2024 Dec 5;11:1473696. doi: 10.3389/fvets.2024.1473696 (PMC11656588; doi:10.3389/fvets.2024.1473696)

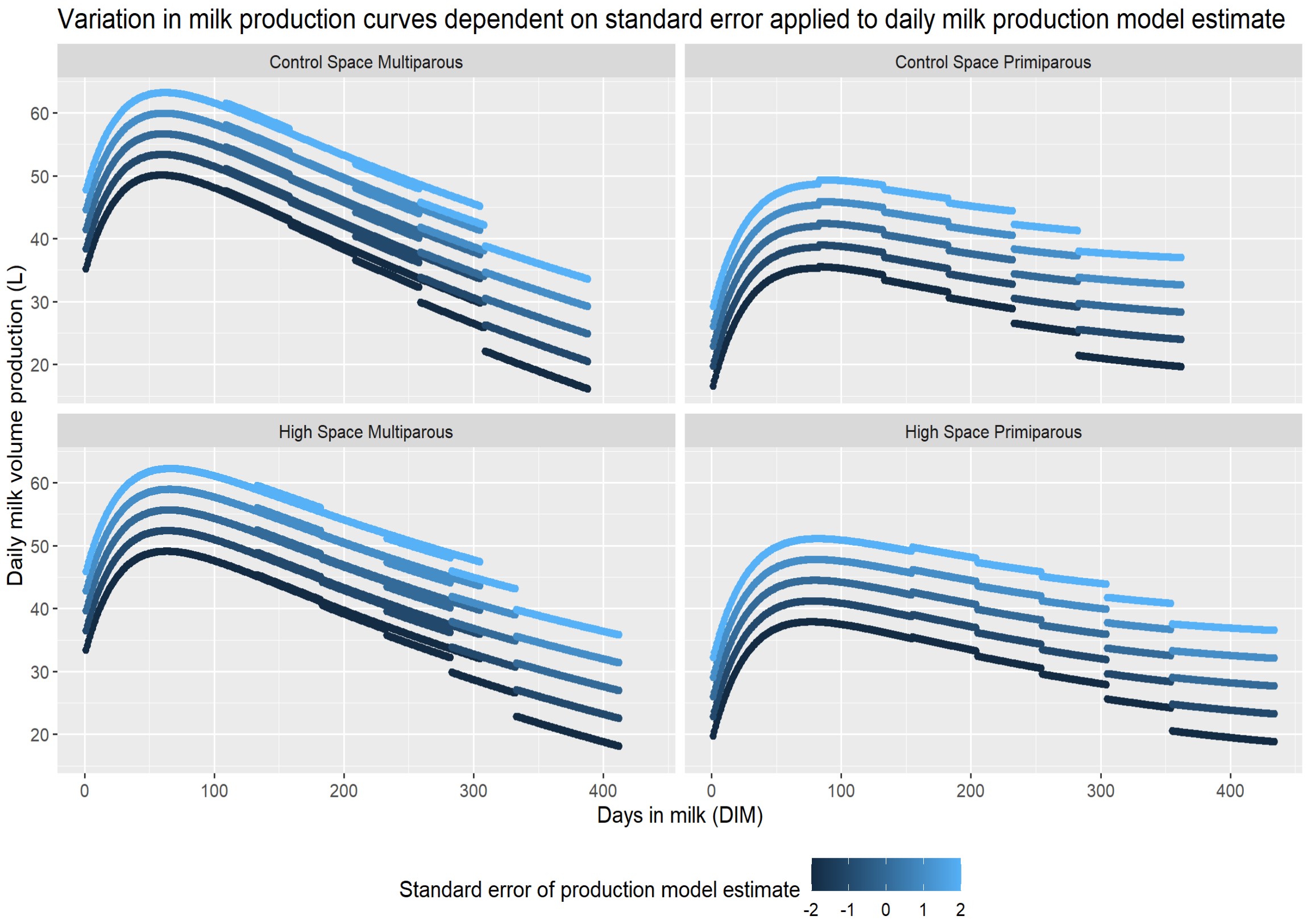

Supplement: SUPPLEMENTARY FIGURE S1 — Figure to show the stochastic variation added to the milk volume production models. As the standard of error increases from negative (dark blue) to positive (light blue) the overall production curve of the simulated cow daily milk production increases (y-axis) and this is also dependent on days in milk (y-axis). This has been split by effect to high space versus control and primiparous versus multiparous lactation curves. [file Image_1.JPEG]
